# An automatic solution making device for quality control of dyestuffs: design philosophy and implementation

**DOI:** 10.1155/S1463924679000547

**Published:** 1979

**Authors:** Hans-Christian Mez, Gero Michel

**Affiliations:** Central Research Dept. Ciba-Geigy Ltd. Basel CH-4002 Switzerland


### An automatic solution making device for quality control of dyestuffs: design philosophy and implementation

**Hans-Christian Mez and Gero Michel,**
*Central Research Dept., Ciba-Geigy Ltd., CH-4002 Basel, Switzerland.*

### Introduction

Dyestuff producers try to supply their customers with products whose quality must remain virtually unchanged over a number of years. This requirement accounts for the extreme importance of quality control in the dyestuff manufacturing process. The dyestuff quality control laboratory has to ensure that any deviations of the products from the standard with respect to the colour parameters (i.e. strength, brilliance and hue) are recognised and quantified in a reliable and reproducible manner.

The classical method of dyestuff colour assessment is based on the application of samples to textile substrates (e.g. strands of yarn or pieces of cloth) under standard conditions and subsequent visual comparison of the results with a number of standard substrate samples dyed under identical conditions. This procedure is both time and labour consuming and consequently expensive. Moreover, it depends entirely on the know-how and expertise of qualified colourists, and the final result is always a subjective judgement.

For reasons of economy and reliability, a less time-consuming and more objective method would be desirable. Fortunately, a mathematical model that, in many cases, correlates optical transmission data measured on dyestuff solutions with colour assessment obtained on substrates dyed with the sample dyestuff has been developed and described by Spahni [1]. This model makes it possible to predict, with adequate accuracy, the results of an eventual classical colour assessment without actually dyeing a substrate.

Such a method is particularly useful for the numerous intermediary tests that lead to a marketable dyestuff formulation. The final products of our dyestuff division are all assessed in the classical manner.

Bypassing the tedious dyeing process offers an economic opportunity that should not be offset by use of a time-consuming and error-prone procedure for the preparation of dyestuff solutions of precisely known concentrations. As a logical consequence it was decided that the entire quality control procedure—that is preparation of dyestuff solutions, spectrophotometric measurements, computation of the predicted value and computation of possible corrective measures—should be performed automatically.

### Problem definition

Figure 1 summarizes the principal steps required for fully automatic quality control by the solution method.

In the present paper it is intended to describe only the hardware and software developed for the automatic preparation of dyestuff solutions. Starting from the manual procedure the authors will sketch some of the problems encountered in adapting it to automatic performance and point out some of the actual implementation features which they believe to be of general interest.

### Steps in manual preparation of dyestuff solutions

1. Set-up procedures : Preparation of sample, solvents, beakers, pipettes, etc. and table-search for dissolution prescription (DP).
2. Weighing of dyestuff sample. Sample weight is prescribed in DP, but must be adjusted to varying colour strengths at different stages of the production process.
3. Addition of prescribed amount of primary solvent to sample (The need for a two step dissolution procedure is explained under point 6. of the next paragraph).
4. Dissolution of sample in primary solvent by vigorous stirring and shaking.
5. Removal of a prescribed aliquot from the primary solution after dissolution.
6. Addition of prescribed amount of secondary solvent to the aliquot.
7. Mixing of secondary solution and transfer of a sample to the spectrophotometer.

*Figure 1 Synopsis of automatic quality control of dyestuffs by the solution method.*

| Materials/Data | Procedure | Tools |
|---|---|---|
| Dyestuff Sample ↓ | Dissolution in solvents | Solution Making Device (sample balance, solvent dispenser, solution homogenizer, minicomputer for control). |
| Sample Solution ↓ | Transmission measurements | Spectrophotometer |
| Spectral Data ↓ | Computation | Minicomputer programmed with the coloristic model, data base with calibration data for dyestuffs of interest. |
| Colorimetric Interpretation ↓ | Computation | Same as for previous procedure |
| Coloristic Correction Proposal | | |

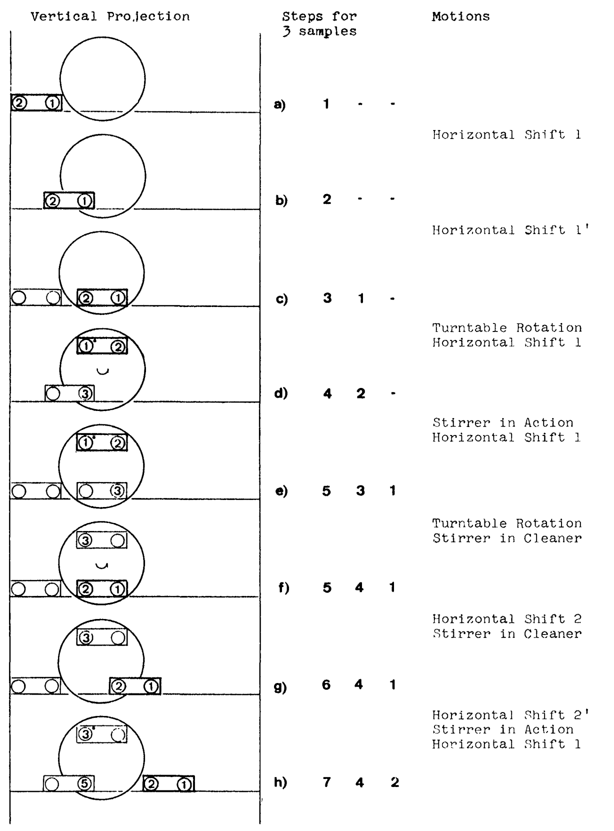

*Figure 2 SMD sequence scheme.*

### Basic design considerations for the automatic preparation of dyestuff solutions for photometric measurement and automatic assessment

A number of specifications for a solution making device (SMD) result from the nature of the samples and the method of assessment:

1. Because production dyestuffs are not homogeneous a minimum sample size of 1 g is required.
2. Non-linearities in the assessment model and the restricted optimum sensitivity range of the spectrophotometer call for solution concentrations deviating no more than 5% from a standard value which may vary from about 5 mg/1 to 300 mg/1.
3. The amount of solution available for spectrophotometry should be at least 50 ml to allow adequate washing of the flow-through cell to prevent cross-contamination.

Additional specifications arise from economic and operational requirements typical of a quality control laboratory:

4. Since the production process depends on the results of the laboratory any unnecessary delays must be avoided. Solutions must be prepared and assessed as fast as possible without loss of accuracy.
5. For the same reasons the SMD must be capable of handling samples of entirely different colours and properties on a first-in first-out basis in a random sequence.
6. Economy in the use of solvents and ecological considerations call for preparation of the dyestuff solutions in two steps to meet both the criteria for sample size 1. and solution concentration 2.
7. The SMD must be able to handle up to 80 samples during an eight hour day with only one non-technical worker operating it.

### Primary implementation considerations

1. Sample handling must be as simple and undemanding as possible. Automatic precision weighing of an adequate amount of sample relieves the operator of a tedious task.
2. The sample size of 1 g or more and the relatively low solubility of a number of dyestuffs necessitate the use of about 500 ml of solvent for the primary solutions.
3. The primary solutions must be thoroughly mixed and an aliquot taken for further dilution.
4. The amount of solvent required for preparation of the secondary solution must be accurately calculated and added to the aliquot.
5. The true amount of solvent added must be determined and the actual final concentration calculated.
6. Every weighing and dispensing step must be performed with an accuracy of 0.1% or better to ensure a final accuracy of better than 1% for the solution concentration.

Obviously, a means for the precise dispensing of solvents and for subsequent determination of the true amounts dispensed is required. We decided to use gravimetry rather than volumetry because it provides a positive check on the actual amounts dispensed.

Since the range of the dyestuff sample balance is only 20 g we need a second balance with at least 500 g range for solvent dispensing.

The automatic performance of the various calculations for intermediate and final concentrations calls for a programmable device. This same device will be able to control the hardware. At the time of design (1973) this device was necessarily a mini computer.

### Implementation of the automated dissolution procedure

### Hardware

The automated procedure must explicitly handle a number of intermediate steps implicit in the manual procedure. Thus, provisions must be made for the transfer of material and data between individual steps and for the optimal sharing of available hardware among samples.

As previously pointed out two solutions have to be prepared. The weighing sequence is the same for both solutions, i.e. first a few grams on the one balance and then some 50–500 g on the other balance. The preparation of the primary solution occurs between the two similar weighing sequences. An elegant and efficient solution to the transfer problem was found. It is based on the use of a polyvinyl chloride tray carrying two beakers for the two solutions. The tray can be moved horizontally on a track in the SMD and carries a reflective tag for identification purposes. Transfer of the beakers to the balances in the SMD is done by shifting the tray into the appropriate position over the balances and lifting the entire base-plate which carries the two balances. Pistons on the balances enter the tray through holes in its bottom and lift the beakers which can then be weighed. The distance between the balances' pistons is the same as that between the two beakers in a tray. Transfer from the weighing position to the position where the primary solution is homogenized is performed by rotating a turntable carrying the sample tray through 180°. This turntable is located over the balance base-plate.

Figure 2 shows different stages in a sample's progress through the SMD which will now be explained in detail.

Step numbers (1–7) refer to the manual procedure previously outlined.

a. Set-up (1), includes positioning of a tray with two beakers on the input track and data entry to the computer through a terminal keyboard. On completion of data entry the tray is automatically advanced to the next position.

b. For step (2) the balance base-plate is lifted, putting beaker 1 on balance 1 where it is tared. The operator weighs the dyestuff sample into beaker 1 checking the weight from the balance interface display and releasing control to the computer by pressing a push-button light switch "EIN-WAAGE" on the control panel when the weight is approximately the amount prescribed.
The net sample weight is stored in the computer, and the tray is transferred to the next position by lowering the balances and shifting the tray. The operator may proceed to enter data for the next sample at any time hereafter.

c. For step (3) the balances are again lifted, with beaker 1 and the dyestuff sample now on balance 2. It is tared and about 450 g of primary solvent are added under balance and computer control. The weight is stored and used to calculate the concentration of the primary solution. Transfer to the next position is done by lowering the balances and rotating the turn-table through 180° horizontally.

d. The stirrer is transferred from the cleaning position to the working position in beaker 1 and its motor switched on. Stirring, step (4), continues for a time specified in step (1).

e. Towards the end of the stirring cycle, when the solution is sufficiently mixed, a sample is extracted by a peristaltic pump and stored, in correspondence with step (5). At the end of the stirring cycle the stirrer is removed from beaker 1 and inserted into the cleaning device where it is subjected to an elaborate cleaning procedure. The turn-table is rotated through another 180° after removal of the stirrer, bringing beakers 1 and 2 back to the weighing position.

f. The balances are lifted, beaker 2 is tared on balance 1, and an amount of primary solution sufficient to make about 80 g of secondary solution is dispensed from the aliquot store and weighed, completing step (5). The balances are then lowered and the tray shifted to the next position. During these operations, the stirrer is undergoing the cleaning procedure so that an eventual second sample would have to wait in position (d) until the stirrer is available.

g. The balances are lifted with beaker 2 and the aliquot now on balance 2. The amount of solvent 2 necessary to attain the requisite final concentration is calculated and automatically dispensed under balance and computer control, step (6). The weights are stored. The balances are lowered and the tray is shifted off the turn-table onto the output track.

h. The final concentration of the secondary solution in beaker 2 is calculated and printed on a teletype printer (TTY) alongside the tray identification number. Transfer

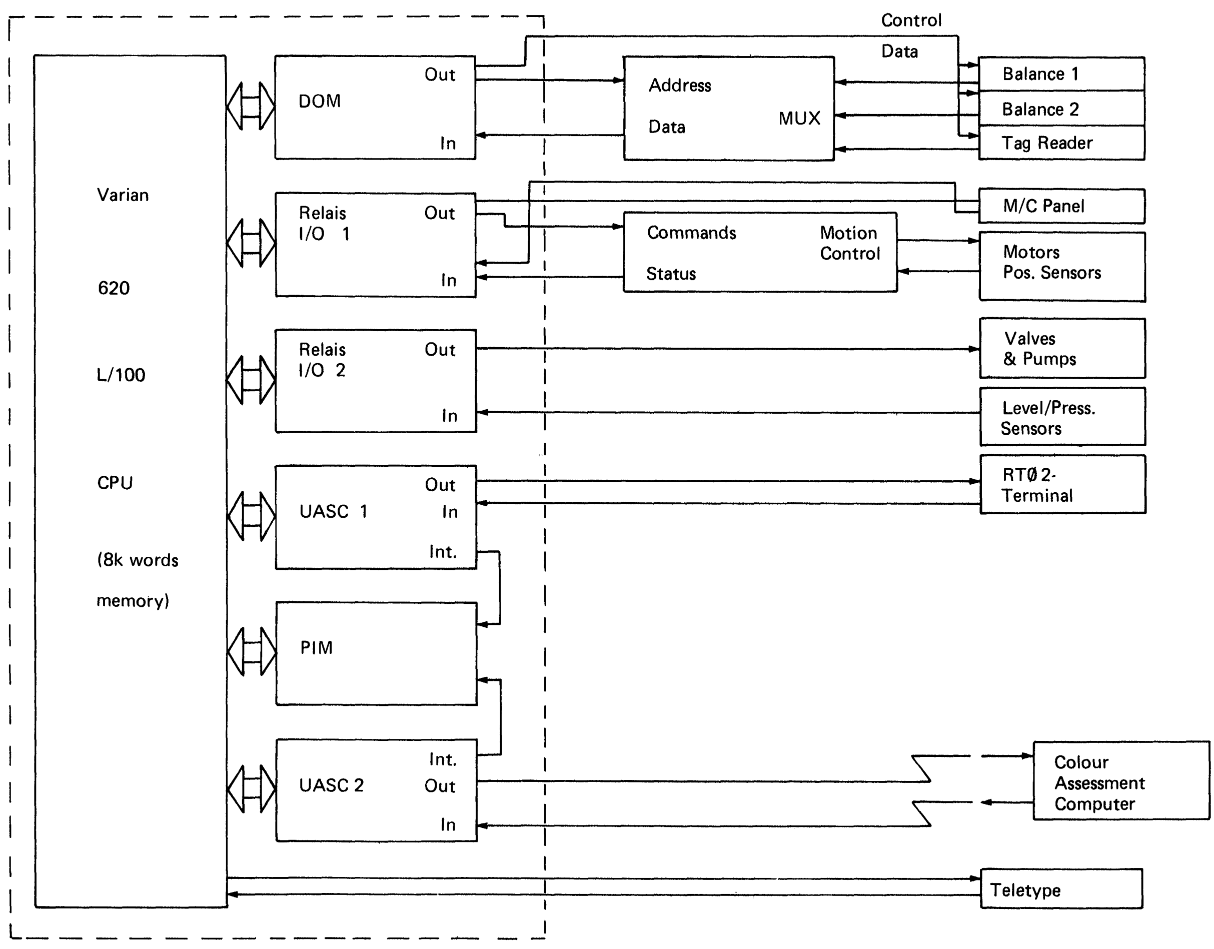

*Figure 3 SMD computer hardware.*

of the solution into the spectrophotometer is done through a Teflon probe inserted into beaker 2 after manual shaking of the latter, step (7).

The spectrophotometer is controlled by the colour assessment computer.

### Hardware details

Only a few of the more generally interesting features of the hardware will be reported.

The accuracy requirements were met by using two types of electronic top loading balances. Balance 1, on which step (b) and (f) are performed, is a Mettler PE 162 with a resolution of 1 mg over a range of 20g. Balance 2, used in steps (c) and (g), is a Mettler PE 1200 with a resolution of 0.01 g in the range −100 to +100 g, and of 0.1 g in the range −1000 to +1000 g.

Not unexpectedly, there were problems because of static electrical charges on the beakers and the sensitivity of the PE 162 balance to air currents in the laboratory. They were overcome by use of aluminium beakers with only the inside coated with Teflon and by a Perspex hood enclosing most of the apparatus.

It was found that a total of six solvents is sufficient to prepare solutions of a large percentage of the production dyestuffs which are amenable to the present method. Two solvents are available for the primary solution, and the secondary solution is made with one of the four remaining solvents. Primary solvents were selected on the basis of their ability to dissolve dyestuffs quickly and quantitatively at room temperature. The criteria for secondary solvents were complete dissolution and optimum correspondence between the transmission spectra and reflection spectra obtained from dyed substrate samples.

The presence of additives in production dyestuffs posed a number of problems in the stirring step. On the one hand, the stirring process should be as vigorous as possible to ensure a short dissolution time; on the other hand, some of the additives have surface activating properties that lead to foaming with subsequent solution spilling. These problems were only overcome after lengthy experiments leading to an elaborate stirrer design that minimizes the amount of air sucked into the solution.

The avoidance of cross-contamination is extremely important because even a few drops of dye solution carried into a subsequent sample could lead to unacceptable results. The cleaning procedure was experimentally optimized with regard to time and solvent consumption. The stirrer and the tubes through which the primary solution sample is extracted and transferred to beaker 2 are washed several times with water and alcohol; cross-contamination is less than the spectrophotometer's sensitivity, even for worst case examples, e.g. yellow after blue or violet.

### Computer hardware

The computer hardware used to control the SMD is illustrated in Figure 3. The mini computer is a Varian 620-L/100 model with 8192 16-bit words core memory. The box on the left of Figure 3 surrounds the components which are located in the Varian 620 chassis and expansion box and were supplied by Varian Data Machines.

The following explanations refer to components in the drawing starting at the top.

The digital output module (DOM) is used to obtain digital data from the two electronic balances and the tag reader. Data are entered through a digital multiplexer (MUX) built by our electronics group.

The Relais I/O Interface 1 serves to light and check push-button light switches on the manual control (M/C) panel and for movement control, i.e. starting and stopping motors for the various transfer devices, and input of the position sensor signals to the computer.

The Relais I/O Interface 2 activates valves and pumps for solution dispensing and handling and transfers level and pressure sensor signals to the computer.

There are two universal asynchronous serial controllers (UASC) capable of interrupting the program on signal input through the priority interrupt module (PIM).

UASC 1 handles the RTϕ2 terminal used for data entry in a dialogue mode.

UASC 2 controls the communication with the colour assessment computer. The latter is another Varian 620-L/100 mini computer with 16384 words of core memory programmed to acquire the spectral data and transform them to colour assessments.*

A Teletype (TTY) is directly linked to the Varian 620 central processing unit (CPU). It is used for documentation of input parameters and output of the final concentration. Optionally, it will also list intermediate results for checking purposes.

### Software

Software is divided into three categories:

Hardware handling software (HHS), problem oriented software (POS), and system software (SS). Their various purposes and means of implementation will be discussed in the following paragraphs.

### Hardware handling software

As the name implies, HHS is that part of software designed to allow the computer to operate the hardware which was previously described and illustrated in Figure 3. It is made up of a set of subroutines that interact with the real world environment of the SMD. Typical HHS routines perform such tasks as

- read the status of position, level and pressure indicators through one of the two Relais I/O cards,
- activate sample transfer devices, such as the turntable, the various horizontal tray shifts or the balance base-plate, by generating appropriate output to the motion control electronics,
- open or close valves for solvent dispensing,
- read digital BCD data from the balances or the tag reader through the MUX, etc.

HHS is tailored to the specific hardware and restricted to systems using the same type of peripheral hardware on the same type of computer. HHS is generally written in assembly language. (Computer manufacturers generally supply HHS for standard peripherals, e.g. disk or printers, as so-called drivers.)

### Problem oriented software

POS comprises routines that perform tasks characteristic for the process or procedure that are automated. They are fairly independent of the details handled by HHS. As an example, Figure 4 shows a flow diagram of medium resolution for step (a) in Figure 2.

The actual implementation of the sub-task 'Light push-button switch (pbs) "RTϕ2 INPUT" is done by a subroutine call to one of the HHS routines, specifically CALL R1ON, PBS1. Note that it is unnecessary to know how subroutine R1ON works as long as the correct parameter is passed to it.

Typical POS routines perform such tasks as

- support the question and answer dialogue for parameter input on the RTϕ2 terminal, and check answers for validity,
- control the solvent dispensing process through a feed-back loop,
- communicate with the colour assessment computer which serves as a data base for dissolution prescriptions and, when on-line to the SMD, keeps track of a sample's progress and waits for the final concentration and the solution,

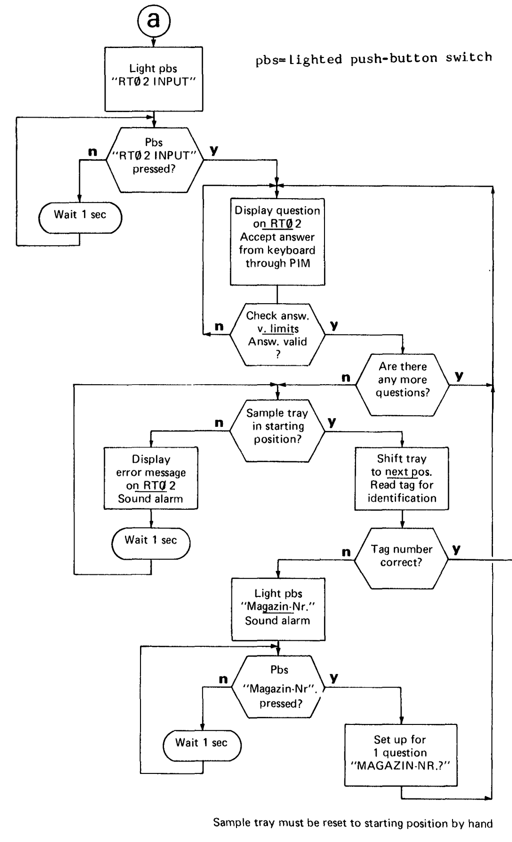

*Figure 4 SMD flow diagram for set-up routine.*

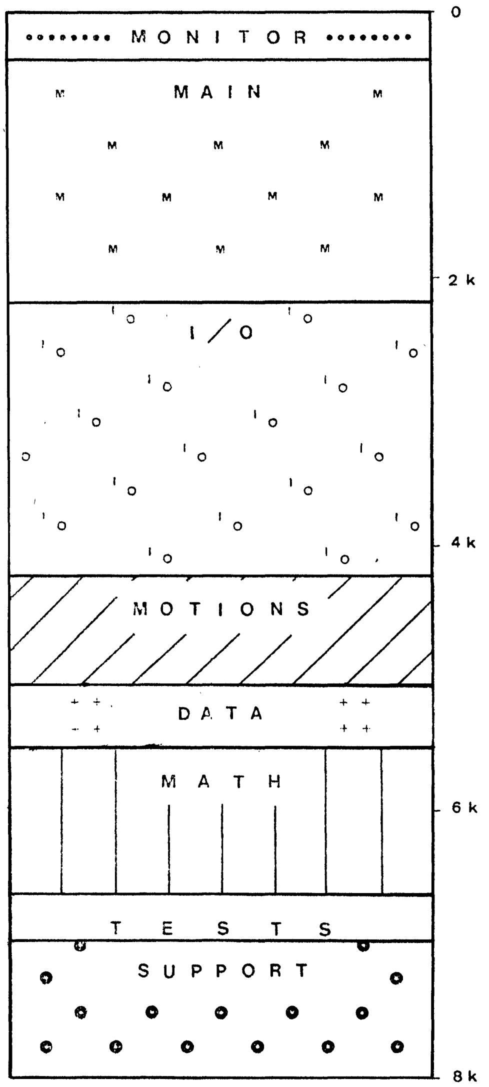

*Figure 5 SMD computer core allocation.*

– carry out various computations involving weights and solution concentrations, etc.

A large portion of POS has the extremely important function of ensuring that no hardware collisions will occur and that error conditions will be adequately handled. Thus in Figure 4, the positioning of the sample tray is checked before the horizontal shift mechanism is activated, and absence of a positive check will result in the display of an error message on the RTϕ2 terminal. Moreover, an intermittent alarm will be sounded and the check repeated every second until the tray has been set to the proper position. After a positive check additional checks will be run before the horizontal tray shift is activated. The balance baseplate must be in the lower position, the turntable must be in one of its two initial positions and no other sample tray must obstruct the path of the tray. The first two tests are done by HHS routines, the third one, however, requires a book-keeping scheme which will be dealt with in the paragraph on system software (SS).

POS is often written in higher-level languages, such as FORTRAN or BASIC. In the SMD, however, assembly language was used for maximum efficiency and core economy. In order to obtain readable and transparent code extensive use was made of subroutines.

### System software

System software enables the computer's central processing unit (CPU) to execute POS and HHS routines in an orderly manner by allocation of CPU time and of hardware access to individual tasks. System software is essentially independent of the other categories of software. Its structure and complexity are directly related to the amount of interaction between individual routines that must be co-ordinated. A batch processing computer may run on fairly simple system software, whereas a real-time system requires rather elaborate routines to handle interrupts and run several tasks quasi-concurrently.

For the SMD a fixed sequence of routines is clearly inadequate. None of the real-time monitors available from Varian Data Machines in 1975 was considered satisfactory and it was therefore decided to design and implement original system software.

The foremost design criteria were reliability, transparency and core economy. The work load on the CPU is low and only a limited number of events require fast (real-time) response. These are handled by the priority interrupt module. All other events are polled regularly from the tasks running under a time schedule. Thus, the monitor system is both event and time controlled. Monitor clock resolution was chosen at 10 msec to obtain sufficiently short response times without undue system overhead.

For successful handling by the monitor the problem-oriented software must be logically divided into a number of

**Table 1. Routines in the SMD monitor**

| Routine | Parameters | Function | Control on Exit |
|---|---|---|---|
| TIMO | – | Job scheduler: runs through task table and starts first active job with starting time ⩾ TNOW | Active Job/TIMO |
| ZABL | – | Reads monitor clock, transfers value to TNOW | Calling Routine |
| IDLE | – | Suspends calling task for 0.2 sec. | TIMO |
| SUSP | NSEC | Suspends calling task for NSEC sec. | TIMO |
| SUSP | –NSEC, ADDR | Suspends calling task for NSEC sec, sets restart address to ADDR | TIMO |
| STOP | NSEC | Stops calling task by changing status to 'dormant', task can only be restarted after NSEC sec. | TIMO |
| STOP | –NSEC, ADDR | Stops calling task by changing status to 'dormant', task can only be restarted after NSEC sec, restart address is set to ADDR. | TIMO |
| BRCH | ADDR | Branches calling task to address ADDR (Differs from normal jump because TIMO is activated) | TIMO |
| PAUS | NCS | Halts calling task for NCS/100 sec, without returning control to TIMO. Useful for tasks that must keep CPU and/or peripheral control. | Calling Routine |
| IFOF | MADD | Checks contents of memory address MADD: If (MADD) = ϕ, calling task continues else, test is repeated once every second until satisfied | Calling Routine/TIMO |

tasks. One of these is the main program which controls a sample's progress through the SMD. The other tasks handle input/output procedures and supervise the activation of transfers or the entire cleaning routine for the solution stirrer. A task is defined in the monitor context as a closed POS routine that may call any other tasks or routines. It can be either active or dormant. A dormant task can only be activated by an active task, whilst an active task can stop only itself but no other task.

Allocation of CPU time to the tasks is controlled by a task table with four consecutive memory words reserved for each task. Word 1 reflects the status of the task (active or dormant), words 2 and 3 indicate the time at which an active task should next be allocated CPU time (resolution 10 msec), and word 4 contains the address at which program execution is to resume.

An interesting aspect of the system monitor is that three different samples in the SMD at the same time can be controlled by only one copy of the main program. The trick is to generate three independent task table entries for the three samples which address different portions of the same routine. In this way the three samples will be run through the SMD completely asynchronously at the maximum possible speed.

The need for a book-keeping system to avoid hardware collisions has already been mentioned. The same problem exists when a task attempts to call a routine or task that is currently used by another task; the problem is aggravated by the outdated sub-routine call structure of the Varian 620 computer which impedes the writing of re-entrant code. The book-keeping system used here relies on software 'flags', i.e. bits set in specific memory words that are checked before the critical programs are activated. Each task sets or resets only those bits that correspond to its task number.

The monitor was implemented in assembly language. It comprises 8 routines and occupies only 203 words of core memory. The interrupt routine that updates the system clock every 10 milli-seconds and the routines that set and reset the flag bits according to the task number occupy another 65 and 29 memory words, respectively. The task table which controls 13 tasks requires 53 memory words.

A short summary of the monitor routines is given in Table 1.

The final core lay-out is presented in Figure 5. Note that one eighth of the available core was reserved for the Varian support programs AIDII and BLD for convenient program debugging. If needed this space would be available for additional POS or HHS routines. The mathematics routines occupy a full 14% of the available memory while the monitor needs only 2.5%.

ACKNOWLEDGMENTS

We thank Dr. H. Heuberger and H. Seitzinger for suggesting the problem and for their continuing effort in the practical use of the apparatus.

We are indebted to H.R. Schatzmann and H.P. Stolz, as well as to R. Hofer and A. Wüthrich for their persistent and creative contributions to all phases of the project.

REFERENCE

[1] Spahni, H., 'A new method for the instrumental color-control of dyestuffs' in *Proceedings 3rd AIC Congress: Colour 77.* 1977 Adam Hilger, London, paper C13.

